# Supplementary material for: Light-induced engagement of microglia to focally remodel synapses in the adult brain
Source: eLife. 2020 Aug 18;9:e58435. doi: 10.7554/eLife.58435 (PMC7470825; doi:10.7554/eLife.58435)
Supplement: Transparent reporting form [file elife-58435-transrepform.docx]

***eLife’s* transparent reporting form**

We encourage authors to provide detailed information *within their submission* to facilitate the interpretation and replication of experiments. Authors can upload supporting documentation to indicate the use of appropriate reporting guidelines for health-related research (see [EQUATOR Network](http://www.equator-network.org/%20)), life science research (see the [BioSharing Information Resource](https://biosharing.org/)), or the [ARRIVE guidelines](http://www.plosbiology.org/article/info:doi/10.1371/journal.pbio.1000412) for reporting work involving animal research. Where applicable, authors should refer to any relevant reporting standards documents in this form.

If you have any questions, please consult our Journal Policies and/or contact us: [editorial@elifesciences.org](mailto:editorial@elifesciences.org).

**Sample-size estimation**

- You should state whether an appropriate sample size was computed when the study was being designed
- You should state the statistical method of sample size computation and any required assumptions
- If no explicit power analysis was used, you should describe how you decided what sample (replicate) size (number) to use

Please outline where this information can be found within the submission (e.g., sections or figure legends), or explain why this information doesn’t apply to your submission:

This information is present in the Materials and Methods section. Sample size planning is described in the methods section and was estimated based on previous studies (Miyamoto A. et al. ,2016; Wake H. et al. , 2009 and Reshef R. et al., 2017). In these studies, between 4-7 mice were used per group in order to find structural changes of spines after the contact with microglia or after the treatment with PLX3397. For this study we needed a minimal total number of 12 animals to detect differences between the control and treated group with 80% power and a significance level alpha of 0.05 (G*Power version 3.0.10, Medium effect size f of 0.25, F tests-ANOVA: Pre-Posttest, within-between interaction). Additionally, 20% of animals was added to select mice with good cranial windows, which resulted in the total number of 14 mice.

**Replicates**

- You should report how often each experiment was performed
- You should include a definition of biological versus technical replication
- The data obtained should be provided and sufficient information should be provided to indicate the number of independent biological and/or technical replicates
- If you encountered any outliers, you should describe how these were handled
- Criteria for exclusion/inclusion of data should be clearly stated
- High-throughput sequence data should be uploaded before submission, with a private link for reviewers provided (these are available from both GEO and ArrayExpress)

Please outline where this information can be found within the submission (e.g., sections or figure legends), or explain why this information doesn’t apply to your submission:

The information related to the biological replicates and how they were obtained is described in the Materials and Methods section. The unit of observation (mouse) for this study is reported in each figure legend as well as the number of biological replicates per mouse. Treatment of the data is described in Materials and Methods, Statistical analysis header.

**Statistical reporting**

- Statistical analysis methods should be described and justified
- Raw data should be presented in figures whenever informative to do so (typically when N per group is less than 10)
- For each experiment, you should identify the statistical tests used, exact values of N, definitions of center, methods of multiple test correction, and dispersion and precision measures (e.g., mean, median, SD, SEM, confidence intervals; and, for the major substantive results, a measure of effect size (e.g., Pearson's r, Cohen's d)
- Report exact p-values wherever possible alongside the summary statistics and 95% confidence intervals. These should be reported for all key questions and not only when the p-value is less than 0.05.

Please outline where this information can be found within the submission (e.g., sections or figure legends), or explain why this information doesn’t apply to your submission:

Statistical analysis methods are described in Materials and Methods section, raw data for each graph is provided with this submission. In each figure legend, the statistical test is mentioned as well as the centers and error bars definitions. Exact P-values are described in each figure legend for both significant and non-significant results.

(For large datasets, or papers with a very large number of statistical tests, you may upload a single table file with tests, Ns, etc., with reference to sections in the manuscript.)

**Group allocation**

- Indicate how samples were allocated into experimental groups (in the case of clinical studies, please specify allocation to treatment method); if randomization was used, please also state if restricted randomization was applied
- Indicate if masking was used during group allocation, data collection and/or data analysis

Please outline where this information can be found within the submission (e.g., sections or figure legends), or explain why this information doesn’t apply to your submission:

Animals were allocated to the treatment groups by simple randomization method. Blinding was implemented during group allocation. This information is provided in the Material and Methods section.

**Additional data files (“source data”)**

- We encourage you to upload relevant additional data files, such as numerical data that are represented as a graph in a figure, or as a summary table
- Where provided, these should be in the most useful format, and they can be uploaded as “Source data” files linked to a main figure or table
- Include model definition files including the full list of parameters used
- Include code used for data analysis (e.g., R, MatLab)
- Avoid stating that data files are “available upon request”

Please indicate the figures or tables for which source data files have been provided:

A summary table with means, s.e.m. and P-values for each studied parameter and raw data for the generation of graphs are presented with this submission. A data availability statement is provided in the article file: “All measurements, statistical analyses and the R code generated and used in this study are included in the manuscript”.
